# Global Air Quality and Health Co-benefits of Mitigating Near-Term Climate Change through Methane and Black Carbon Emission Controls

**DOI:** 10.1289/ehp.1104301

**Published:** 2012-03-14

**Authors:** Susan C. Anenberg, Joel Schwartz, Drew Shindell, Markus Amann, Greg Faluvegi, Zbigniew Klimont, Greet Janssens-Maenhout, Luca Pozzoli, Rita Van Dingenen, Elisabetta Vignati, Lisa Emberson, Nicholas Z. Muller, J. Jason West, Martin Williams, Volodymyr Demkine, W. Kevin Hicks, Johan Kuylenstierna, Frank Raes, Veerabhadran Ramanathan

**Affiliations:** 1 U.S. Environmental Protection Agency, Washington, DC, USA; 2 Department of Environmental Health, Harvard School of Public Health, Boston, Massachusetts, USA; 3 NASA Goddard Institute for Space Studies and Columbia Earth Institute, Columbia University, New York, New York, USA; 4 International Institute for Applied Systems Analysis, Laxenburg, Austria; 5 European Commission, Joint Research Centre, Ispra, Italy; 6 Stockholm Environment Institute, Environment Department, University of York, York, United Kingdom; 7 Department of Economics, Middlebury College, Middlebury, Vermont, USA; 8 Environmental Sciences and Engineering Department, Gillings School of Global Public Health, University of North Carolina–Chapel Hill, Chapel Hill, North Carolina, USA; 9 Environmental Research Group, King’s College London, London, United Kingdom; 10 United Nations Environment Programme, Nairobi, Kenya; 11 Scripps Institution of Oceanography, University of California–San Diego, San Diego, California, USA

**Keywords:** air quality, climate change, health impact analysis, outdoor air, particulate matter

## Abstract

Background: Tropospheric ozone and black carbon (BC), a component of fine particulate matter (PM ≤ 2.5 µm in aerodynamic diameter; PM_2.5_), are associated with premature mortality and they disrupt global and regional climate.

Objectives: We examined the air quality and health benefits of 14 specific emission control measures targeting BC and methane, an ozone precursor, that were selected because of their potential to reduce the rate of climate change over the next 20–40 years.

Methods: We simulated the impacts of mitigation measures on outdoor concentrations of PM_2.5_ and ozone using two composition-climate models, and calculated associated changes in premature PM_2.5_- and ozone-related deaths using epidemiologically derived concentration–response functions.

Results: We estimated that, for PM_2.5_ and ozone, respectively, fully implementing these measures could reduce global population-weighted average surface concentrations by 23–34% and 7–17% and avoid 0.6–4.4 and 0.04–0.52 million annual premature deaths globally in 2030. More than 80% of the health benefits are estimated to occur in Asia. We estimated that BC mitigation measures would achieve approximately 98% of the deaths that would be avoided if all BC and methane mitigation measures were implemented, due to reduced BC and associated reductions of nonmethane ozone precursor and organic carbon emissions as well as stronger mortality relationships for PM_2.5_ relative to ozone. Although subject to large uncertainty, these estimates and conclusions are not strongly dependent on assumptions for the concentration–response function.

Conclusions: In addition to climate benefits, our findings indicate that the methane and BC emission control measures would have substantial co-benefits for air quality and public health worldwide, potentially reversing trends of increasing air pollution concentrations and mortality in Africa and South, West, and Central Asia. These projected benefits are independent of carbon dioxide mitigation measures. Benefits of BC measures are underestimated because we did not account for benefits from reduced indoor exposures and because outdoor exposure estimates were limited by model spatial resolution.

Tropospheric ozone and black carbon (BC), a component of fine particulate matter (PM ≤ 2.5 µm in aerodynamic diameter; PM_2.5_), have been associated with deleterious effects on human health (e.g., Jerrett et al. 2009; Laden et al. 2006; Pope et al. 2002), agriculture (e.g., Ashmore 2005), and climate (e.g., Ramanathan and Carmichael 2008). Methane, a relatively short-lived greenhouse gas (residence time 8–10 years), is an ozone precursor that affects background ozone concentrations. Controlling methane emissions may be a promising means of simultaneously mitigating climate change and reducing global ozone concentrations, compared with controlling shorter-lived ozone precursors [nitrogen oxides (NO_x_), carbon monoxide (CO), and non-methane volatile organic compounds (NMVOCs)] (West et al. 2006, 2007). The latter may have larger and more immediate air quality and health benefits near the areas with emission reductions but smaller benefits (CO, NMVOC) or net disbenefits (NO_x_) for climate. Major anthropogenic sources of methane include fossil fuel production and distribution, landfills, livestock, rice cultivation, and wastewater treatment. BC is a product of incomplete combustion from sources such as biomass burning, transportation (mainly diesel vehicles), residential combustion, and industry, and is coemitted with other pollutants, including NO_x_, NMVOCs, CO, sulfur dioxide (SO_2_), and organic carbon. Climate benefits of reducing BC may be partially offset by associated reductions of coemitted pollutants that may have a net cooling effect on climate (and a net warming effect when reduced), either directly (organic carbon) or after chemical transformation in the atmosphere (organic carbon, SO_2_, and NO_x_). However, all emission reductions leading to reduced ozone and PM_2.5_ concentrations would be expected to have health benefits.

Mitigating ozone and BC may benefit climate and health simultaneously (e.g., Jacobson 2002; Smith et al. 2009; West et al. 2006); because methane and BC are short-lived relative to the long-lived greenhouse gases [e.g., carbon dioxide (CO_2_)], mitigation would reduce the rate of climate change in the near-term (Jackson 2009; Ramanathan and Carmichael 2008). Although a recent series of studies has examined the ancillary health benefits of greenhouse gas mitigation (Haines et al. 2009), the health benefits of mitigating ozone and BC as climate forcers have been studied less extensively. Studies examining the health impacts of all fossil fuel and biofuel emissions (Jacobson 2010), percentage reductions in ozone precursors (West et al. 2006) and BC (Anenberg et al. 2011), and adoption of European vehicle emission standards in the developing world (Shindell et al. 2011) suggest that controlling methane and BC emissions may substantially benefit global public health, particularly in Asia where large populations are exposed to high PM_2.5_ and ozone concentrations (Ramanathan et al. 2008).

The United Nations Environment Programme (UNEP) and the World Meteorological Organization (WMO) therefore initiated an integrated assessment of the potential climate, health, agricultural, and economic benefits that would be achieved by further implementing methane and BC mitigation measures already employed in various parts of the world (UNEP 2011). In the present study, we used emissions scenarios developed for the UNEP/WMO assessment to examine the potential air quality and health benefits of methane and BC mitigation measures in more detail.

## Methods

*Emission scenarios and modeling*. We used five emissions scenarios developed for the UNEP/WMO assessment to examine methane and BC mitigation impacts on air quality and health globally and in five world regions [see Supplemental Material, Figure 1 (http://dx.doi.org/10.1289/ehp.1104301)]. These scenarios include a present-day (2005) reference case, a 2030 reference scenario that incorporates International Energy Agency energy projections (International Energy Agency 2009) and all presently agreed upon (but no additional) policies affecting emissions (see Supplemental Material, Table 2 and Figure 2), and three different policy scenarios in which varying degrees of additional emission controls are implemented by 2030. To isolate the impacts of anthropogenic emission changes, all scenarios assume identical meteorology and natural emissions [including open biomass burning (i.e., wildfires); year 2000]. The emission scenarios and their projected effects on climate are detailed by Shindell et al. (2012) and are summarized in Supplemental Material, pp. 4–9.

**Figure 1:**
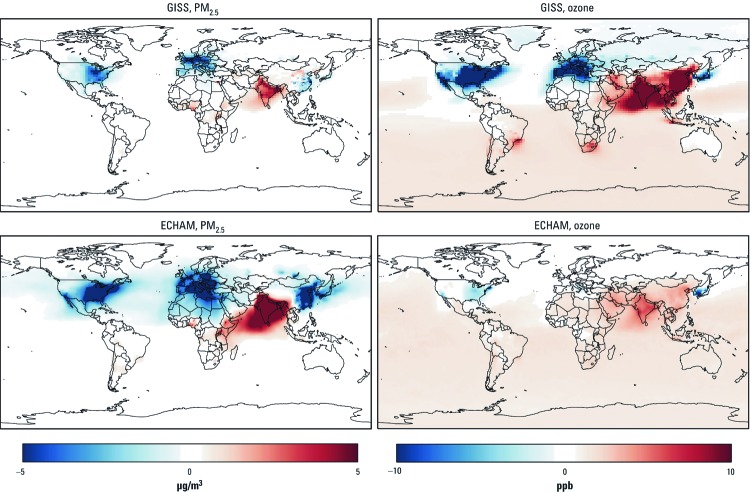
Estimated changes in annual average PM_2.5_ (µg/m^3^) and seasonal (6‑month) average 1-hr daily maximum ozone (ppb) concentration for the 2030 reference scenario relative to 2005, based on the GISS and the ECHAM models.

**Table 1:** Description of the 14 methane and BC mitigation measures included in the three increasingly stringent policy scenarios for 2030.

Scenario	Mitigation measure
Methane measures: technical measures for methane emissions		Extended pre-mine degasification and recovery and oxidation of methane from ventilation air from coal mines
		Extended recovery and use—rather than venting—of associated gas and improved control of unintended fugitive emissions from the production of oil and natural gas
		Reduced gas leakage from long-distance transmission pipelines
		Separation and treatment of biodegradable municipal waste through recycling, composting, and anaerobic digestion as well as landfill gas collection with combustion/utilization
		Upgrading primary wastewater treatment to secondary/tertiary treatment with gas recovery and overflow control
		Control of methane emissions from livestock, mainly through farm-scale anaerobic digestion of manure from cattle and pigs
		Intermittent aeration of continuously flooded rice paddies
BC group 1: technical measures for reducing emissions of incomplete combustion		Diesel particle filters as part of a Euro VI package for road and off-road diesel vehicles
		Introduction of clean-burning stoves for cooking and heating in developing countries
		Replacing traditional brick kilns with vertical shaft kilns and Hoffman kilns
		Replacing traditional coke ovens with modern recovery ovens, including the improvement of end-of-pipe abatement measures in developing countries
BC group 2: nontechnical measures to eliminate the most polluting activities		Elimination of high-emitting vehicles in road and off-road transport (excluding shipping)
		Ban of open field burning of agricultural waste
		Substitution of clean-burning cook stoves using modern fuels for traditional biomass cook stoves in developing countries

**Figure 2:**
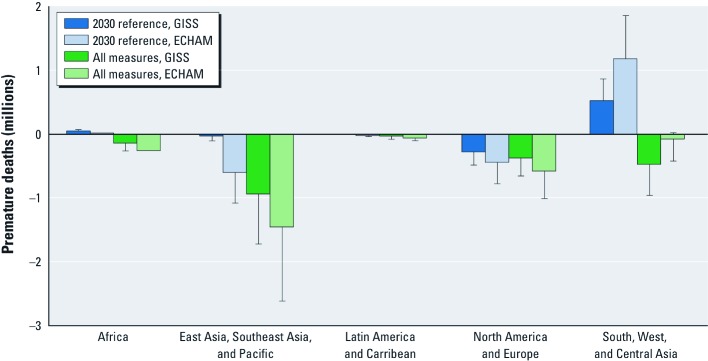
Estimated changes in premature PM_2.5_-related mortality (cardiopulmonary and lung cancer deaths) and ozone-related mortality (respiratory deaths) for the 2030 reference scenario and assuming implementation of methane plus BC group 1 and BC group 2 (all) measures relative to 2005, based on 2030 population projections. 95% CIs reflect uncertainty in the CRF only.

We selected the three policy scenarios based on an evaluation of the potential climate impacts of approximately 2,000 mitigation measures defined in the International Institute for Applied Systems Analysis (IIASA) Greenhouse Gas and Air Pollution Interactions and Synergies (GAINS) model (Amann et al. 2011). Climate impacts of each measure were classified according to CO_2_ equivalence, which was calculated based on global warming potential (GWP) over a 100-year time horizon for predicted methane, CO, SO_2_, NO_x_, NMVOCs, BC, organic carbon, and CO_2_ emission changes following implementation of the control measure (Shindell et al. 2012). Based on this evaluation, we identified 14 individual methane and BC control measures that would achieve approximately 90% of the climate benefits feasible for all of the evaluated measures combined (according to the CO_2_ equivalence metric). The 14 measures were grouped into three increasingly stringent policy scenarios for 2030 [Table 1; see also Supplemental Material, pp. 4–8 (http://dx.doi.org/10.1289/ehp.1104301)]. The first scenario includes seven technological measures for controlling methane emissions. The second adds four technological measures (BC group 1) for reducing emissions of incomplete combustion, including implementation of Euro 6 and Euro VI equivalent vehicle emission standards (requiring installation of diesel particulate filters) (European Union 2010, 2011) and improving traditional biomass cook stoves in developing countries. We assumed that emission factors for cook stoves would decline in all regions to levels consistent with emissions from rocket stoves, resulting in a 25% decrease in BC and 80–90% decreases in other species, including organic matter, CO, NMVOC, methane, and direct PM_2.5_, relative to emissions from traditional stoves (MacCarty et al. 2008). Realistically, emission reductions from cookstoves could be lower depending on stove adoption and use; however, other stove technologies may also be more effective at lowering emissions. Finally, the third and most stringent policy scenario adds three regulatory measures (BC group 2) to eliminate high-emitting vehicles, biomass cook stoves (in developing countries), and agricultural waste burning.

**Table 2:** Global simple and population-weighted (Pop-wt) average reductions in annual average PM_2.5_ (µg/m^3^) and maximum 6‑month average 1-hr daily maximum ozone (ppb) concentrations, avoided PM_2.5_ cardiopulmonary and lung cancer deaths and ozone respiratory deaths (millions), and avoided YLL (millions) based on 2030 population projections for increasingly stringent mitigation policies relative to the baseline scenario for 2030.

Methane measures			Methane and BC group 1 measures			Methane, BC group 1, and BC group 2 measures		
Result	PM2.5	Ozone	PM2.5	Ozone	PM2.5	Ozone
Simple average												
GISS		–0.01		3.08		0.15		5.34		0.22		5.66
ECHAM		–0.03		3.60		0.18		4.00		0.27		3.92
Pop-wt average												
GISS		–0.03		2.82		2.90		9.95		3.98		11.0
ECHAM		–0.12		4.09		3.59		4.96		4.92		4.71
Avoided deaths												
GISS		–0.02 (–0.01, –0.03)		0.07 (0.02, 0.11)		1.39 (0.46, 2.47)		0.28 (0.09, 0.47)		1.93 (0.63, 3.48)		0.31 (0.10, 0.52)
ECHAM		–0.06 (–0.02, –0.11)		0.10 (0.03, 0.17)		1.74 (0.57, 3.12)		0.13 (0.04, 0.21)		2.42 (0.78, 4.40)		0.12 (0.04, 0.20)
Avoided YLL												
GISS		–0.12 (–0.04, –0.21)		0.61 (0.20, 1.01)		11.8 (3.85, 21.0)		2.54 (0.82, 4.28)		16.2 (5.25, 29.3)		2.81 (0.90, 4.74)
ECHAM		–0.59 (–0.20, –1.01)		0.94 (0.31, 1.56)		14.9 (4.86, 26.6)		1.15 (0.38, 1.92)		20.5 (6.63, 37.4)		1.06 (0.35, 1.76)
95% CIs (shown in parentheses) reflect uncertainty in the CRFs for PM2.5- and ozone-related mortality only. Estimates are based on simulations using the GISS and ECHAM models.												

We simulated ozone and PM_2.5_ concentrations using two global composition-climate models, the NASA Goddard Institute for Space Studies (GISS) model for Physical Understanding of Composition-Climate INteractions and Impacts (GISS-PUCCINI; Shindell et al. 2006), and the ECHAM-HAMMOZ model (Pozzoli et al. 2008), referred to here as GISS and ECHAM. We assumed that mitigation measures would be fully implemented and their impacts on concentrations fully realized by 2030. Methane concentrations (accounting for chemical and biological loss processes) were averaged over years 15–19 of each simulation to realize the steady-state effects of methane reductions, although additional minor impacts may occur beyond this period. GISS has a horizontal resolution of 2° latitude × 2.5° longitude with 40 vertical layers from the surface to 0.1 hectopascal (hPa). ECHAM has a horizontal resolution of 2.8° × 2.8° and 31 vertical layers up to 10 hPa. Both models simulate BC, organic carbon, SO_4_, sea salt, and dust. GISS also includes nitrate (NO_3_). We multiplied simulated organic carbon concentrations by 1.4 to estimate total organic matter concentrations (Cooke et al. 1999). Using a different conversion factor would affect organic matter concentrations proportionally. Because these coarse model resolutions cannot capture fine concentration gradients, particularly for primary PM_2.5_ species (BC and organic carbon) around urban areas, we allocated BC and organic carbon to 0.5° × 0.5° resolution according to population density, following Shindell et al. (2011; see their Supplemental Information). All other species, including ozone, SO_4_, and NO_3_, were simply regridded to 0.5° × 0.5° resolution, because secondary pollutants are generally more spatially homogeneous. For the main results, we excluded dust and sea salt (which are assumed to be natural) and use the health impact function described below. We also examined the sensitivity of mortality results to inclusion of dust and sea salt and to different magnitudes and shapes of the health impact function.

*Health impact assessment.* We used epidemiologically derived health impact functions to estimate changes in premature PM_2.5_- and ozone-related mortality between the 2030 reference scenario and 2005, and between the 2030 reference scenario and the three policy scenarios individually, using 2030 population projections for all scenario comparisons to isolate the impacts of simulated concentration changes. We assumed log-linear relationships between PM_2.5_ or ozone concentrations and relative risks (RR), following Anenberg et al. (2010), and calculated the fraction of baseline deaths attributable to a given change in concentration (attributable fraction; AF) as

AF = (RR – 1)/RR = 1 – exp^–βΔ^*^X^*, [1]

where β is the concentration–response factor (CRF, the estimated slope of the log-linear relation between PM_2.5_ or ozone concentration and mortality) and Δ*X* is the change in pollutant concentration. We multiplied AF by the baseline mortality rate (*y*_0_) and population size (Pop) to estimate the change in premature deaths (ΔMort) that would result from a given change in concentration (Δ*X*):

ΔMort = *y*_0_ × Pop × (1 – exp^–βΔ^*^X^*). [2]

Because disease survival times vary among populations, we estimated the change in years of life lost (ΔYLL) due to a change in premature deaths using the baseline YLL (YLL_0_) per death:

ΔYLL = ΔMort × YLL_0_/*y*_0_. [3]

We applied Equations 2 and 3 in each 0.5° × 0.5° grid cell using corresponding population sizes, baseline mortality and YLL rates, and the simulated changes in PM_2.5_ and ozone concentrations.

We calculated CRFs for PM_2.5_ based on long-term RR estimates starting from the American Cancer Society (ACS) cohort study (Pope et al. 2002). Specifically, for a 10-µg/m^3^ increase in annual average PM_2.5_, RRs for all-cause, cardiopulmonary disease, and lung cancer mortality were 1.06 [95% confidence interval (CI): 1.02, 1.11), 1.09 (95% CI: 1.03, 1.16), and 1.14 (95% CI: 1.04, 1.23), respectively, when averaged based on data for 1979–1983 and 1999–2000. Although the ACS cohort was large compared with other PM_2.5_ cohort studies [e.g., the Harvard Six Cities Study (Laden et al. 2006)], results may underestimate the PM_2.5_–mortality relationship because well-educated affluent populations are overrepresented in the cohort and because exposure was measured with greater error than in other studies. A 2008 expert elicitation (including ACS authors) produced a mean all-cause mortality CRF estimate [approximately 1.1% mortality increase per 1-µg/m^3^ increase in PM_2.5_ (Roman et al. 2008)] that was between the CRFs calculated from the ACS (~ 0.6%) and Harvard Six Cities Study (~ 1.6%) RR estimates. The expert elicitation (Roman et al. 2008), however, did not estimate cause-specific RRs, which may be more applicable globally than all-cause mortality. We therefore multiplied the cause-specific CRFs calculated from the Pope et al. (2002) RR estimates by 1.8, the factor difference between the all-cause CRFs from the expert elicitation mean and Pope et al. (2002). A newer ACS reanalysis reported 40% higher cardiopulmonary effect estimates with tighter confidence intervals for all RR estimates (Krewski et al. 2009), but these results were not available for the expert elicitation. Therefore, we examined the effect of these RRs in a sensitivity analysis only. Other recent cohort studies have reported considerably larger estimated effect sizes than the expert mean judgment (e.g., Miller et al. 2007; Puett et al. 2009), suggesting that our approach is conservative. Although some BC-rich PM_2.5_ mixtures may be more toxic than other mixtures (Maynard et al. 2007; Smith et al. 2009), we assumed that all PM_2.5_ components and mixtures are equally toxic because evidence for differential toxicity is currently inconclusive.

For ozone, we used long-term RR estimates from the ACS cohort (Jerrett et al. 2009) based on a two-pollutant model that controlled for PM_2.5_, in which ozone was significantly associated only with death from respiratory causes. For a 10-ppb increase in the seasonal (6-month) average of 1-hr daily maximum ozone, the RR of respiratory disease was 1.04 (95% CI: 1.010, 1.067). The study by Jerrett et al. (2009) was the first major study to find a significant positive relationship between chronic ozone exposure and mortality in a general population; biological plausibility for this result is supported by evidence from toxicology and human exposure studies showing that ozone affects airway inflammation, pulmonary function, and asthma induction and exacerbation (National Resource Council 2008). Global extrapolation of U.S.-based RR estimates for both PM_2.5_ and ozone is supported by generally consistent short-term PM_2.5_ and ozone mortality relationships around the world (e.g., Health Effects Institute 2010).

We used simulated concentrations in the first model layer for surface concentrations, and used annual average concentrations for PM_2.5_ and the maximum 6-month average of the 1-hr daily maximum for ozone, consistent with the epidemiology studies. We projected population growth (global population is projected to increase to 8.4 billion in 2030) based on the Intergovernmental Panel on Climate Change Special Report on Emissions Scenarios (SRES) B2 scenario, which is near the center of projected population growth estimates for the different SRES scenarios (Intergovernmental Panel on Climate Change 2000). We estimated mortality only for the fraction of the population ≥ 30 years of age to be consistent with the age range of the ACS cohort, and we used present-day baseline mortality and YLL rates from the World Health Organization as described previously by Anenberg et al. (2010).

## Results

*Impacts of the future reference scenario.* Both the GISS and ECHAM models indicated that PM_2.5_ and ozone concentrations would change dramatically, and with great spatial variability around the world, in the 2030 reference scenario relative to baseline estimates for 2005 (Figure 1). Projected concentration changes are solely due to emission changes because meteorology was held constant. Changes in climate would also impact concentrations to a lesser degree (e.g., Jacobson 2008).

We estimated that these concentration changes would substantially affect air pollution-related mortality around the world. Unless otherwise specified, ranges reported for expected changes in mortality and YLL represent the lowest and highest 95% CI bounds estimated using either the GISS or the ECHAM model, where the 95% CIs reflect uncertainty in the CRF. We expect that regulations that are currently in place or planned in North America and Europe will reduce PM_2.5_ and ozone concentrations substantially, resulting in 0.1–0.8 million avoided PM_2.5_-related deaths per year (0.5–4.8 million YLL) in 2030, with the majority of avoided deaths in Europe [Figure 2; see also Supplemental Material, Figures 4 and 5 (http://dx.doi.org/10.1289/ehp.1104301)]. Regulations are also expected to reduce PM_2.5_ concentrations in East Asia, Southeast Asia, and the Pacific, resulting in 0.1–1.1 million avoided PM_2.5_-related deaths (0.4–7.7 million YLL) annually, based on 2030 population projections. However, we estimated that increased ozone concentrations in East Asia, Southeast Asia, and the Pacific would cause 0–0.2 million additional premature ozone-related deaths (0.1–1.4 million YLL) per year. In addition, increased PM_2.5_ and ozone concentrations in South, West, and Central Asia resulting from rapid emissions growth would cause an estimated 0.1–1.8 million (1.2–15.9 million YLL) additional PM_2.5_-related premature deaths and 0–0.2 million (0.1–2.4 million YLL) additional ozone-related premature deaths annually.

**Figure 3:**
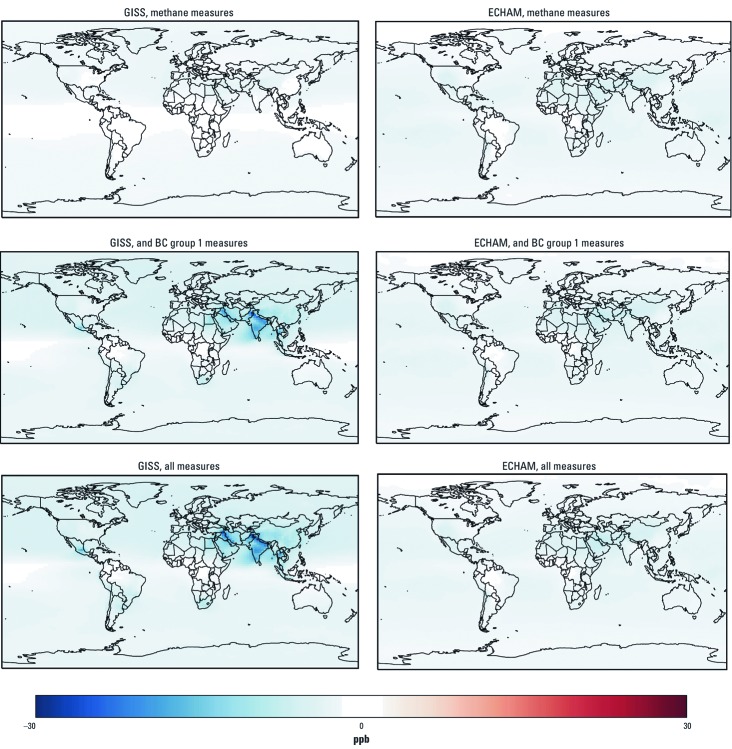
Estimated changes in seasonal (6‑month) average 1-hr daily maximum ozone concentration (ppb) in 2030 for successive implementation of methane measures, methane plus BC group 1 measures, and methane plus BC group 1 and BC group 2 (all) measures, relative to the 2030 reference scenario, based on the GISS and the ECHAM models.

**Figure 4:**
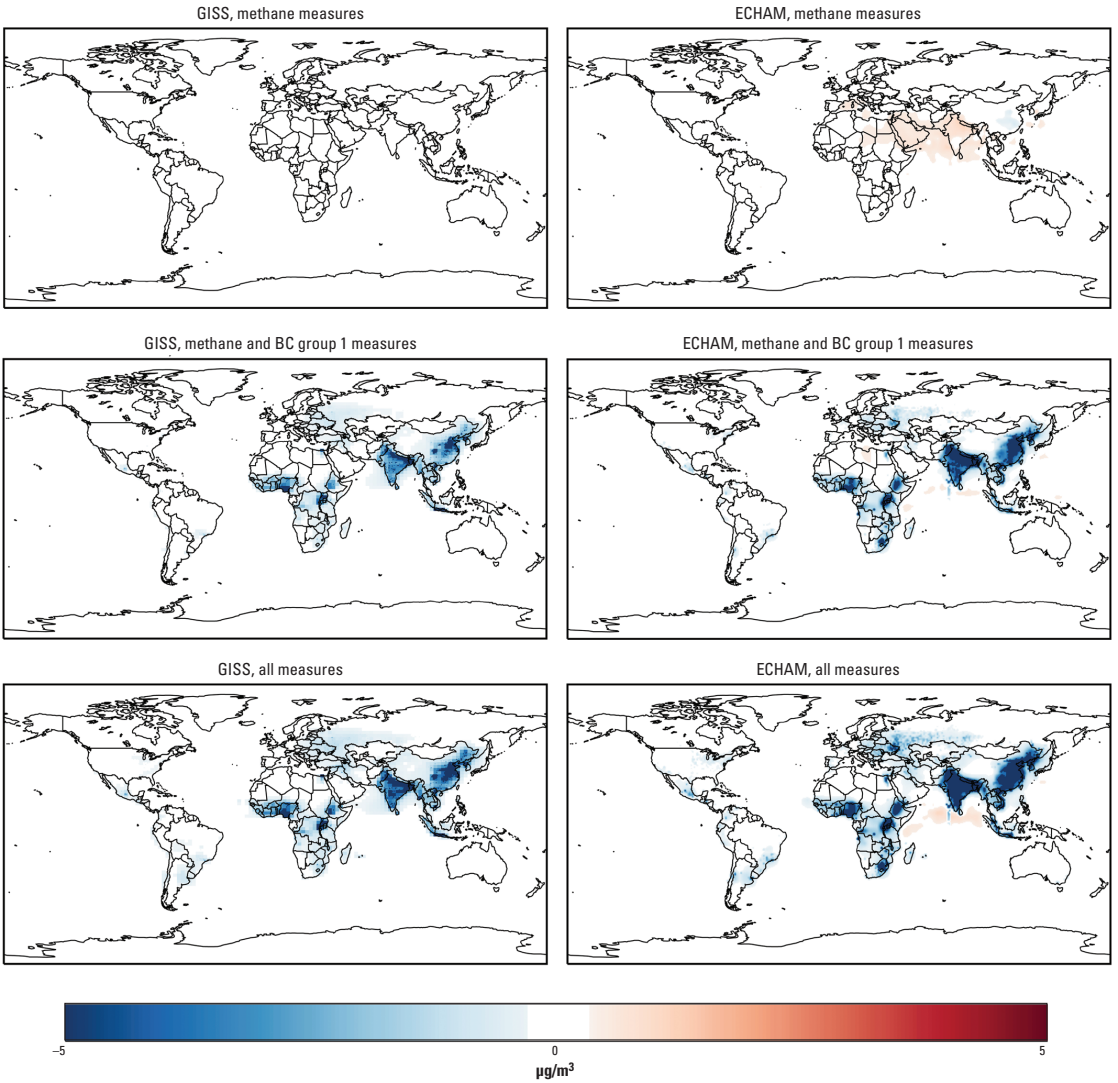
Estimated changes in annual average PM_2.5_ concentration (µg/m^3^) in 2030 for successive implementation of methane measures, methane plus BC group 1 measures, and methane plus BC group 1 and BC group 2 (all) measures, relative to the 2030 reference scenario, based on the GISS and the ECHAM models.

*Benefits of the mitigation measures.* Relative to the 2030 reference scenario, implementing the methane measures (Table 1) would decrease seasonal (6-month) average 1-hr daily maximum ozone concentrations by 3–4 ppb (Table 2 and Figure 3). Projected ozone concentrations decreased fairly evenly across the globe due to the relatively longer lifetime of methane compared with other ozone precursors (e.g., NO_x_, VOCs). However, simulated annual average PM_2.5_ concentrations increased slightly from northern Africa to the Indian subcontinent in response to the methane measures due to particle formation resulting from changes in oxidant concentrations (Table 2 and Figure 4), as demonstrated previously by West et al. (2006). However, when BC and methane measures were applied together, these increases were projected only by the ECHAM model and were limited to a small area off the coast of eastern Africa and India. Adding the BC measures would reduce population-weighted PM_2.5_ concentrations by 4–5 µg/m^3^ compared with the 2030 reference scenario. Adding BC measures would also decrease ozone concentrations due to reductions in coemitted ozone precursors, but GISS projected larger reductions (11 ppb reduction when methane and BC measures were applied together) than did ECHAM (5 ppb reduction). Projected reductions in ozone concentrations resulting from the BC measures were localized near the emissions sources (primarily in South and East Asia where emissions are largest) because of the short atmospheric lifetime of the ozone precursors that are affected by the BC measures [NO_x_ and CO; see Supplemental Material, Figure 3 (http://dx.doi.org/10.1289/ehp.1104301)]. Spatial patterns of simulated concentration changes were similar for both models, but GISS projections for ozone were more sensitive to precursors that would be affected by BC measures, whereas ECHAM projected greater reductions in ozone in response to the methane measures and greater reductions in PM_2.5_ in response to BC measures.

**Figure 5:**
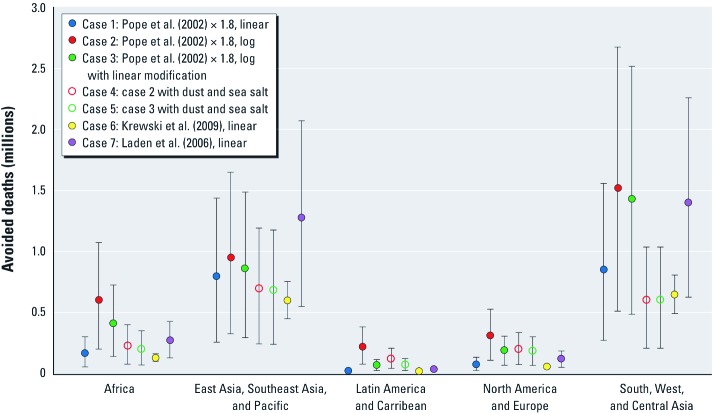
Estimated annual PM_2.5_-related cardiopulmonary and lung cancer deaths assuming implementation of methane plus BC group 1 and BC group 2 (all) measures relative to the 2030 reference scenario using concentrations simulated by the GISS model and different assumptions for the CRF, based on 2030 population projections.

We estimated that implementing all measures would avoid 0.6–4.4 million PM_2.5_-related deaths (5.3–37.4 million YLL) and 0.04–0.52 million ozone-related deaths (0.35–4.7 million YLL) in 2030 [Table 2; see also Supplemental Material, Figures 6–9 (http://dx.doi.org/10.1289/ehp.1104301)]. For both models, > 80% of the estimated mortality benefits from implementation of all three groups of measures would occur in Asia, where large populations are exposed to high concentrations (Table 3). BC groups 1 and 2 measures (four technological measures for reducing emissions of incomplete combustion and three nontechnical measures to reduce the most polluting activities, respectively) would account for 72% and 26% of avoided deaths globally for either model. In contrast, estimated global mortality benefits of the methane measures were an order of magnitude smaller than those of the BC measures (approximately 2%), because of reductions of non-methane ozone precursor and organic carbon emissions associated with implementation of the BC measures and because of stronger relationships of PM_2.5_ with mortality. The estimated contribution of each policy measure to the total mortality benefit in each region generally followed the global contributions. When low-carbon CO_2_ measures (decrease in use of fossil fuel) were included in both the reference and policy scenarios, estimates showed approximately 10% fewer avoided deaths in East Asia, Southeast Asia, and the Pacific and in South, West, and Central Asia [see Supplemental Material, Figure 10 (http://dx.doi.org/10.1289/ehp.1104301)]. Implementing the methane and BC measures would reduce mortality substantially in all regions, and in some regions (Africa and South, West, and Central Asia) would reverse trends of increasing mortality due to air pollution (Figure 2).

**Table 3:** Distributions of estimated numbers of avoided premature deaths according to policy measures and world regions, relative to the 2030 reference scenario.

Percent of avoided deaths attributed to each group of policy measuresa					Percent of all avoided deaths resulting from implementation of policy measuresb				
Region	Methane	BC Group 1	BC Group 2	Methane	Methane and BC Group 1	Methane, BC Group 1 and BC Group 2
Global												
GISS		2.36		72.09		25.55						
ECHAM		1.62		72.09		26.29						
Africa												
GISS		3.62		74.36		22.01		12.77		8.71		8.32
ECHAM		2.78		72.88		24.34		17.84		10.68		10.40
East Asia, Southeast Asia, and Pacific												
GISS		2.22		68.52		29.26		38.14		38.48		40.50
ECHAM		6.29		64.27		29.45		130.84		32.34		33.79
Latin America and Caribbean												
GISS		9.67		64.36		25.96		7.37		1.79		1.80
ECHAM		12.0		54.71		33.26		13.72		1.68		1.85
North America and Europe												
GISS		6.53		68.76		24.70		11.94		4.36		4.31
ECHAM		3.81		60.63		35.56		12.29		4.58		5.24
South, West, and Central Asia												
GISS		1.56		75.50		22.94		29.78		46.66		45.08
ECHAM		–2.49		79.23		23.26		–74.69		50.73		48.72
aThe individual impact of each group of policy measures is estimated based on the difference in mortality with the implementation of the increasingly stringent policy scenarios; the total for each row equals 100%. bProportions of avoided deaths associated with the successive implementation of the policy scenarios; column totals for each model (GISS or ECHAM) equal 100%.												

*Sensitivity analysis.* We examined the effect of varying CRF assumptions on estimated avoided deaths from implementing all methane and BC measures (Figure 5). In the main results (case 1), we excluded dust and sea salt because evidence for toxicity of these components is weaker than that for particulate products of incomplete combustion. Including dust and sea salt would have increased estimated PM_2.5_ concentrations from a maximum of 62–73 µg/m^3^ (in the main results) to a maximum of 269–451 µg/m^3^. Whereas linearity of the CRF has been demonstrated up to 30 µg/m^3^ in the ACS study (Krewski et al. 2009) and up to 40 µg/m^3^ in the Harvard Six Cities study (Laden et al. 2006), some evidence suggests that the PM_2.5_ mortality relationship may flatten at high concentrations (e.g., Pope et al. 2009). We therefore examined several sensitivity cases in which the shape of the CRF was varied. Case 1 represented our baseline assumptions of linear CRFs from Pope et al. (2002) multiplied by 1.8 to scale up to the mean of the expert elicitation (Roman et al. 2008), that is, that cardiopulmonary and lung cancer mortality would increase by 1.6% and 2.4% with each 1-µg/m^3^ increase in PM_2.5_, as in the main results (case 1). For case 2 we used log CRFs from Pope et al. (2002), multiplied by 1.8, such that the slopes of the relation between log-transformed PM_2.5_ concentration and cardiopulmonary and lung cancer mortality, respectively, were 0.2794 and 0.4180 (0.1552 and 0.2322 prior to scaling, as reported by Cohen et al. 2004). Case 3 was identical to case 2, except the log CRFs were modified to be linear below 7 µg/m^3^. Cases 4 and 5 were identical to cases 2 and 3 except they included dust and sea salt in estimated total PM_2.5_ concentrations. Because dust and sea salt were not significantly affected by the mitigation measures, using linear functions with dust and sea salt produced results that were similar to case 1. Two additional sensitivity cases examined the effect of using linear CRFs from the latest ACS reanalysis in which cardiopulmonary and lung cancer mortality increased by 1.3% and 1.4%, respectively, with each 1-µg/m^3^ increase in PM_2.5_ (Krewski et al. 2009; case 6) and linear CRFs from the latest Harvard Six Cities reanalysis in which cardiopulmonary and lung cancer mortality increased by 2.8% and 2.7% with each 1-µg/m^3^ increase in PM_2.5_ (Laden et al. 2006; case 7). The significantly higher RR estimates reported by Laden et al. (2006) are still lower than estimates from other studies with less exposure error (e.g., Puett et al. 2009).

Compared with regional avoided deaths estimated using a linear function, those estimated using log functions without dust and sea salt (case 2) were 1.2–8.3 times higher and had larger differences in the least polluted regions due to a higher marginal impact of PM_2.5_ on mortality for the log functions at low concentrations. When dust and sea salt were included in PM_2.5_ concentrations (case 4), estimates were 12–29% lower in Asia (where PM_2.5_ concentrations are high) and 1.4–4.6 times higher in less-polluted regions. Modifying the functions to be linear at low concentrations (cases 3 and 5) reduced the inflated estimates that occurred in relatively unpolluted regions when log functions were used. Using RR estimates from Krewski et al. (2009; case 6) reduced estimated deaths by approximately 25% relative to the main results. Although RR estimates by Krewski et al. (2009) are higher than those reported by Pope et al. (2002), we multiplied CRFs from Pope et al. (2002) by 1.8 for the main results. Using RR estimates from the Harvard Six Cities cohort (case 7) increased estimates by approximately 60%. Uncertainty ranges were large for each case, with the exception of case 6, because Krewski et al. (2009) estimated more precise RRs than the other studies. However, confidence intervals overlapped among estimates from all of the sensitivity analyses.

## Discussion and Conclusion

We estimated the potential future air quality and health benefits resulting from implementing 14 specific methane and BC emission control measures selected for their near-term climate benefits (Table 1). We estimate that these measures could reduce global population-weighted average surface PM_2.5_ and ozone concentrations by 3.98–4.92 µg/m^3^ (23.0–33.7%) and 4.71–11.0 ppb (6.5–17.0%), respectively, and avoid 0.6–4.4 and 0.04–0.52 million annual premature deaths globally in 2030. More than 80% of the health benefits of these measures are estimated to occur in Asia. Based on our estimates, avoided deaths would represent 1–8% of cardiopulmonary and lung cancer deaths among those ≥ 30 years of age and 1–7% of all deaths for all ages, assuming constant baseline mortality rates. BC mitigation measures would account for approximately 98% of the estimated deaths avoided, because BC mitigation would also reduce emissions of non-methane ozone precursors and organic carbon and because concentration–response relationships are stronger for PM_2.5_ than for ozone. Our estimates are consistent with previous health impact assessments of BC and methane reductions (Anenberg et al. 2011; Shindell et al. 2011; West et al. 2006) after accounting for methodological differences [see Supplemental Material, p. 16 (http://dx.doi.org/10.1289/ehp.1104301)].

We used two global composition-climate models (GISS and ECHAM) to improve confidence in our results, and sensitivity analysis indicated that our results and conclusions are not strongly dependent on assumptions for the CRF. However, we were unable to quantify other uncertainties associated with estimating air pollution mortality on a global scale, including uncertainties in the atmospheric model assumptions and inputs (e.g., emissions) and in estimates of population growth and baseline mortality rates. We applied U.S.-based CRFs globally, despite differences in concentrations, air pollutant mixtures, and exposure and population susceptibility characteristics. We assumed that all PM_2.5_ mixtures are equally toxic, despite some evidence that BC-rich mixtures are more toxic than the average (e.g., Smith et al. 2009). These uncertainties may cause under- or overestimation in the results.

The benefits of implementing BC measures are likely to have been underestimated because we did not account for health benefits of reduced indoor exposure from the burning of solid fuel, which has been estimated to cause 1.6 million premature deaths annually (Smith et al. 2004). In addition, while we downscaled modeled BC and organic carbon concentrations to a finer resolution grid, observed BC concentrations near highly populated regions that rely on biomass combustion for cooking and heating are orders of magnitude higher than the grid mean values used here (Rehman et al. 2011). We also did not consider benefits from reductions in noncarbonaceous primary PM_2.5_ components (e.g., fly ash) that may result from the BC mitigation measures. We estimate that including noncarbonaceous primary PM_2.5_ components would reduce total PM_2.5_ emissions by an additional 18% [see Supplemental Material, Figure 3 (http://dx.doi.org/10.1289/ehp.1104301)] but would have a smaller effect on PM_2.5_ concentration changes (and associated mortality changes), because some PM_2.5_ components included in the PM_2.5_ definition are not emitted directly but are formed in the atmosphere. We did not estimate effects of air pollution on morbidity or infant mortality because of concerns about the quality and availability of concentration–response functions and baseline incidence data globally. We also did not consider health effects of climate change (e.g., direct effects of temperature), which vary across locations and are poorly understood. Finally, we held present-day baseline mortality rates constant to 2030, although economic development around the world is reducing mortality from infectious disease and increasing mortality due to chronic diseases that are more affected by air pollution. Hence the overall health benefits of these interventions are likely to be understated.

The UNEP/WMO assessment demonstrated that further implementation of methane and BC emissions control measures currently employed in some parts of the world can slow the rate of climate change in the decades following implementation (Shindell et al. 2012; UNEP 2011). We conclude that these measures can also substantially benefit global public health, potentially reversing trends of increasing concentrations and air pollution-related mortality in Africa and South, West, and Central Asia. These estimated benefits are independent of CO_2_ mitigation measures. Future research should include both indoor and outdoor concentration changes to quantify the full health and climate benefits of cook stove replacement, and should quantify the benefits and costs of each measure in individual countries or regions to support national-scale policy decisions.

## Supplemental Material

(872 KB) PDF
