# Supplementary material for: Global Air Quality and Health Co-benefits of Mitigating Near-Term Climate Change through Methane and Black Carbon Emission Controls
Source: Environ Health Perspect. 2012 Mar 14;120(6):831–9. doi: 10.1289/ehp.1104301 (PMC3385429; doi:10.1289/ehp.1104301)
Supplement: (872 KB) PDF [file ehp.1104301.s001.pdf]

Supplemental Material:

Global Air Quality and Health Co-Benefits of Mitigating Near-Term Climate Change through Methane and Black Carbon Emission Controls

Susan C. Anenberg, Joel Schwartz, Drew Shindell, Markus Amann, Greg Faluvegi, Zbigniew Klimont, Greet Janssens-Maenhout, Luca Pozzoli, Rita Van Dingenen, Elisabetta Vignati, Lisa Emberson, Nicholas Z. Muller, J. Jason West, Martin Williams, Volodymyr Demkine, W. Kevin Hicks, Johan Kuylenstierna, Frank Raes, Veerabhadran Ramanathan

Table of Contents

Regional definitions 3

Supplemental Material, Figure 1. Regional definitions used in this analysis 3

Selection of the mitigation measures 4

Supplemental Material, Table 1. Values and sources of GWP100s for each species used for the screening of mitigation measures in the GAINS model. 4

Emission changes 5

Supplemental Material, Table 2. Global anthropogenic emissions used in this study for the 2005 and 2030 reference cases, Tg a^{-1}. 6

Supplemental Material, Figure 2. Global anthropogenic emissions used in this study for the 2005 and 2030 reference cases, as in Supplemental Material, Table 2. To compare the emissions magnitudes of the various species on a uniform scale, BC and OC emissions are shown multiplied by 10, methane and CO emissions are divided by 10, and CO_2 emissions are divided by 1000.................................7

Supplemental Material, Figure 3. Percent change in emissions for the successive implementation of methane measures, methane plus BC Group 1 measures, and methane plus BC Group 1 and BC Group 2 measures, relative to the 2030 reference scenario, by region. 8

Supplemental Material, Table 3. Radiative forcing (W/m^2) for the successive implementation of methane measures, methane plus BC Group 1 measures, and methane plus BC Group 1 and BC Group 2 measures, relative to the 2030

reference, calculated by the GISS model. 9

Additional results 10

Supplemental Material, Figure 4. Change in estimated annual premature $PM_{2.5}$ cardiopulmonary and lung cancer and ozone respiratory deaths (lives per 1000 km^2) for the 2030 reference scenario relative to 2005, based on 2030 population. 10

Supplemental Material, Figure 5. Regional change in estimated annual $PM_{2.5}$ cardiopulmonary and lung cancer and ozone respiratory mortality for the 2030 reference scenario relative to 2005, based on 2030 population. Confidence intervals (95%) reflect uncertainty in the CRF only. 11

Supplemental Material, Figure 6. Change in estimated annual premature $PM_{2.5}$ cardiopulmonary and lung cancer and ozone respiratory deaths (lives per 1000 km^2) for the successive implementation of methane measures, methane plus BC Group 1 measures, and methane plus BC Group 1 and BC Group 2 measures, relative to the 2030 reference scenario, based on 2030 population. 12

Supplemental Material, Figure 7. Estimated global annual avoided premature $PM_{2.5}$ cardiopulmonary and lung cancer and ozone respiratory deaths for the successive implementation of methane measures, methane plus BC Group 1 measures, and methane plus BC Group 1 and BC Group 2 measures, relative to the 2030 reference scenario, based on 2030 population. Confidence intervals (95%) reflect uncertainty in the CRF only. 13

Supplemental Material, Figure 8. Regional change in estimated $PM_{2.5}$ cardiopulmonary and lung cancer mortality for the successive implementation of methane measures, methane plus BC Group 1 measures, and methane plus BC Group 1 and BC Group 2 measures, relative to the 2030 reference scenario, based on 2030 population. Confidence intervals (95%) reflect uncertainty in the CRF only. 14

Supplemental Material, Figure 9. Regional change in estimated ozone respiratory mortality for the successive implementation of methane measures, methane plus BC Group 1 measures, and methane plus BC Group 1 and BC Group 2 measures, relative to the 2030 reference scenario, based on 2030 population. Confidence intervals (95%) reflect uncertainty in the CRF only. 14

Supplemental Material, Figure 10. Regional change in estimated $PM_{2.5}$

cardiopulmonary and lung cancer and ozone respiratory mortality for the implementation of all methane and BC mitigation measures relative to the 2030 reference scenario and all methane and BC measures with CO_2 mitigation measures included in both the reference and mitigation scenarios, using concentrations simulated by the GISS model and 2030 population. Confidence intervals (95%) reflect uncertainty in the CRF only. 15

Comparison of results with previous studies..16

References 17

Regional definitions

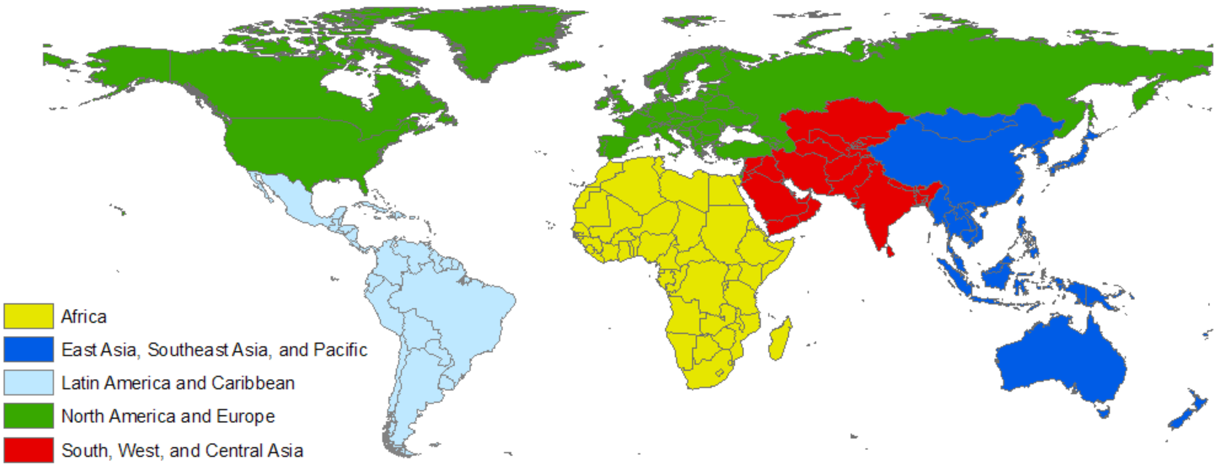

Supplemental Material, Figure 1. Regional definitions used in this analysis.

Selection of the mitigation measures

Most pollutants are not emitted in isolation, but rather in a mixture of species that have different impacts on near-term climate change, some of them heating and others cooling. In addition, most mitigation measures impact the mixture of emissions from a source rather than individual pollutants alone. The overall effect of each mitigation measure depends on the net radiative forcing resulting from changes in emissions of all species. Since the net climate effect can be warming or cooling, we identify a small portfolio of measures with the largest potential reductions in global radiative forcing in 2030. Thus, measures that would increase radiative forcing are excluded from this study.

As described in further detail by the UNEP/WMO Assessment (UNEP 2011) and Shindell et al. (2012), approximately 2000 mitigation measures in 108 world regions in the IIASA GAINS model were screened for their potential climate benefits using the GWP100 metric (see Supplemental Material, Table 1). The measures were then ranked according to their net carbon dioxide equivalence (CO_2eq) accounting for all affected pollutants (i.e., methane, CO, BC, OC, SO_2, NO_x, NMVOCs and CO_2). Results of the GWP evaluation are minimally sensitive to the time horizon since CO_2 emissions were largely unaffected and the effects for all short-lived compounds would change similarly. We found that all measures targeting methane would result in net radiative forcing benefits, while measures to reduce BC differed in estimated net positive or negative climate impacts due to impacts on co-emitted CO and other species. No attempt was made to optimize the selected measures based on health, vegetation, and crop impacts; rather, we simply quantify the health co-benefits of the measures selected for climate benefits.

Supplemental Material, Table 1. Values and sources of GWP100s for each species used for the screening of mitigation measures in the GAINS model (source: UNEP 2011). Note: The GWPs for CO and methane include the indirect effects of ozone.[a]

Species	Mean value	Reference
**CO_2**	1	IPCC (2007)
**CH_4**	25	IPCC (2007)
**CO**	1.9	IPCC (2007)
**VOC**	3.4	IPCC (2007)
**BC**	680	Bond and Sun (2005)
**SO_2**	-40	Fuglestvedt et al. (2009)
**OC**	-69	Fuglestvedt et al. (2009)

[a]GWP100 values represent the radiative forcing of a unit mass of the species given relative to that of CO_2 for a 100 year time horizon.

Emission changes

The 2030 reference scenario assumes significant growth in fossil fuel use relative to 2005, leading to increases in estimated CO_2 (45%) and methane (27%) emissions (See Supplemental Material, Table 2 and Figure 2). However, abatement measures prescribed in current legislation are projected to reduce air pollutant emissions, varying by pollutant and region. While total primary $PM_{2.5}$ emissions (includes non-carbonaceous $PM_{2.5}$, e.g. fly ash) remain approximately constant, BC and OC are estimated to decline by a few percent, mostly due to emission reductions in North America & Europe and Northeast Asia, Southeast Asia & Pacific, where residential coal burning is expected to decline. While projected NO_x (-3%) and SO_2 (-19%) emissions are reduced significantly in North America and Europe, emissions in other regions are expected to grow or remain constant. Asia is projected to contribute most to total $PM_{2.5}$ (>60%), SO_2 (65%), and NO_x (>50%) emissions in 2030.

The methane measures reduce estimated global anthropogenic methane emissions by 38%, with little impact on other species (See Supplemental Material, Figure 3). BC measures together would reduce global anthropogenic BC emissions by 75%, primarily via biomass combustion controls and diesel particle filters. Measures targeting BC would also substantially reduce total primary $PM_{2.5}$ (-50%, of which ~15% is non-carbonaceous, e.g. fly ash), OC (-79%), NO_x (-27%), and CO (-44%). Emissions of non-carbonaceous primary $PM_{2.5}$ components, which we exclude from our $PM_{2.5}$ definition, are projected to be reduced by ~18% of the magnitude of the BC and OC reduction. The BC measures would have little impact on SO_2 emissions since measures reducing sulfate (SO_4; which has a net cooling influence on climate) were largely excluded, as they would not deliver the climate benefit set as a goal in the assessment. Projected emissions are generally reduced most in Asia, followed by Africa. North America and Europe contribute relatively less to emission reductions, except for methane. We also examine the impacts of stabilizing greenhouse gases at 450 ppm of CO_2 equivalent, consistent with a global average temperature increase of ~2° C (IEA 2009). CO_2 measures would reduce SO_2 (-30%) and NO_x (-20%) but have little impact on the other species (~5% decline) since the major sources of CO_2 differ from those of methane and BC.

Supplemental Material, Table 2. Global anthropogenic emissions used in this study for the 2005 and 2030 reference cases, Tg a^{-1}. Source: GAINS model[a], EDGAR v4.1, and Lamarque et al. (2010) for international shipping and aviation (RCP8.5 scenario)

		Region					
**Species**	**Year**	**Africa**	**NE Asia, SE Asia & Pacific**	**Latin America & Caribbean**	**N. America & Europe**	**South, West & Central Asia**	**Global**
**BC**	2005	0.89	1.99	0.35	1.04	1.04	5.46
	2030	1.11	1.76	0.37	0.62	1.37	5.40
**OC**	2005	3.14	5.23	0.90	1.40	2.98	13.80
	2030	3.98	3.86	0.95	1.05	3.20	13.21
**Total $PM_{2.5}$**	2005	6.24	17.04	2.75	5.34	7.68	40.44
	2030	7.70	15.64	2.58	4.57	9.65	40.81
**SO_2**	2005	4.86	37.84	5.04	33.77	14.07	108.64
	2030	3.03	34.39	4.04	16.18	24.18	88.15
**NO_X**[b]	2005	5.08	28.43	8.52	39.43	11.29	114.73
	2030	5.84	35.69	9.11	18.74	21.37	111.19
**CH_4**	2005	34.40	76.58	40.95	82.18	53.79	288.36
	2030	47.77	108.21	52.23	83.21	73.51	365.46
**CO**	2005	75.69	217.67	45.25	176.96	81.89	598.74
	2030	87.27	182.46	39.18	138.32	96.18	544.95
**CO_2**	2005	1.04	9.40	1.46	13.36	3.22	29.91
	2030	1.48	17.04	2.32	13.47	7.35	43.41

[a] Except global NH_3 and NMVOC emissions from solvent use for all countries except Europe, China, and India, that originate from EDGAR v4.1

[b] Reported as NO_2

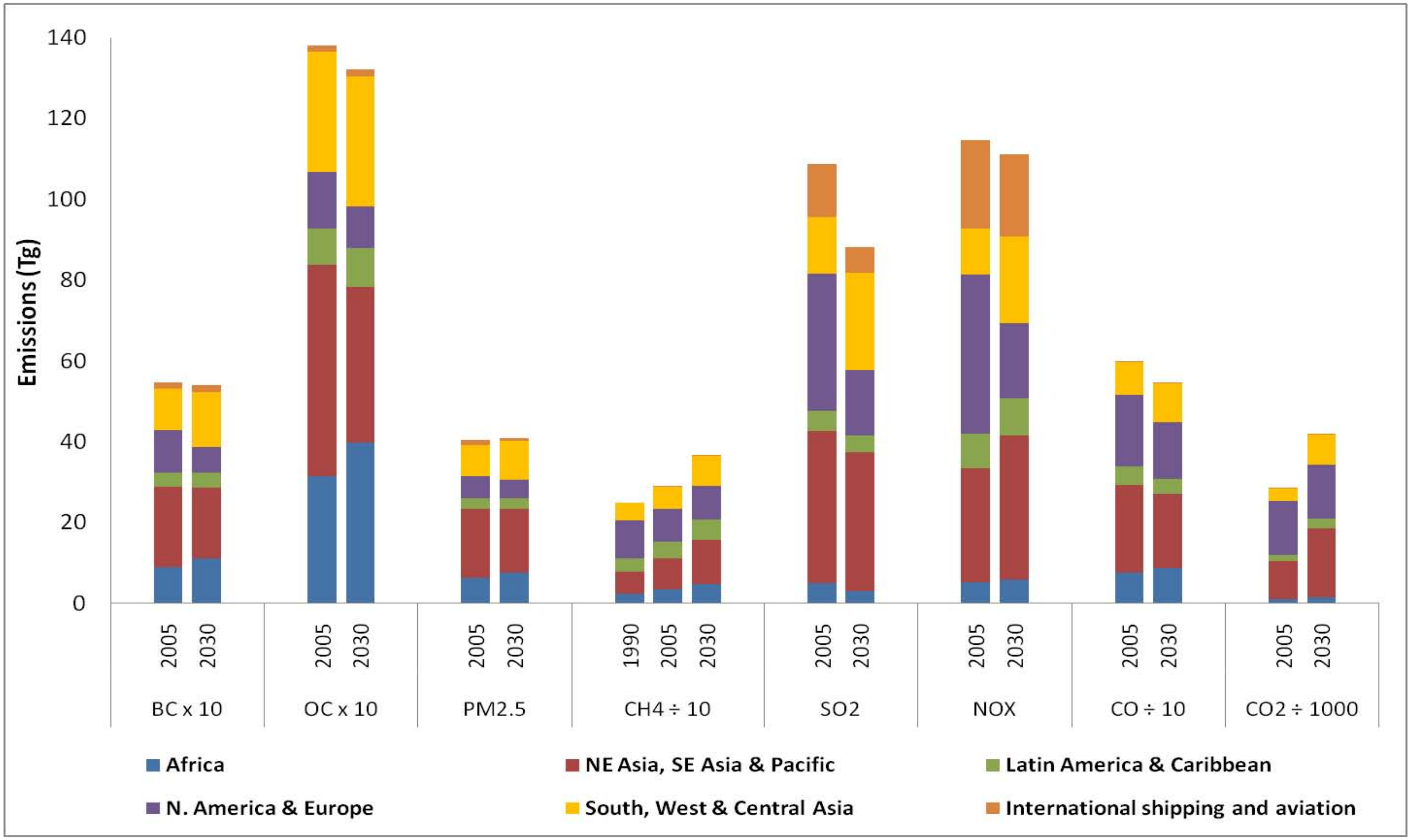

Supplemental Material, Figure 2. Global anthropogenic emissions used in this study for the 2005 and 2030 reference cases, as in Supplemental Material, Table 2. To compare the emissions magnitudes of the various species on a uniform scale, BC and OC emissions are shown multiplied by 10, methane and CO emissions are divided by 10, and CO_2 emissions are divided by 1000.

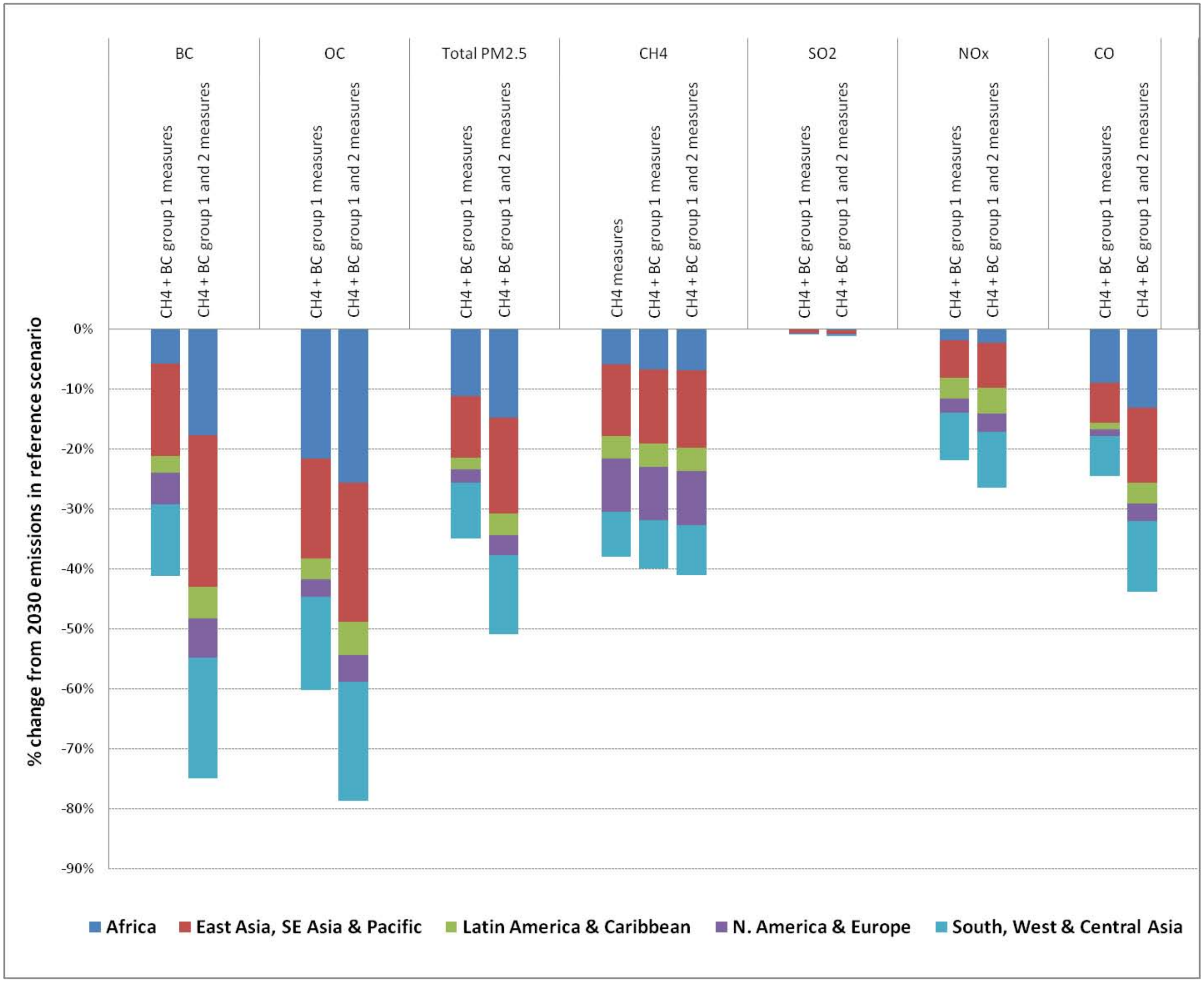

Supplemental Material, Figure 3. Percent change in emissions for the successive implementation of methane measures, methane plus BC Group 1 measures, and methane plus BC Group 1 and BC Group 2 measures, relative to the 2030 reference scenario, by region.

Supplemental Material, Table 3. Radiative forcing (W/m^2) for the successive implementation of methane measures, methane plus BC Group 1 measures, and methane plus BC Group 1 and BC Group 2 measures, relative to the 2030 reference, calculated by the GISS model (source: Shindell et al. 2012).

	**Methane measures**	**Methane + BC Group 1 measures**	**Methane + BC Group 1 + BC Group 2 measures**
**Ozone**	-0.10	-0.17	-0.19
**Methane**	-0.20	-0.20	-0.18
**Aerosols - direct (BC, OC, sulfate, nitrate)**	-0.01 (0.00, 0.00, -0.02, 0.00)	-0.06 (-0.10, 0.06, -0.02, 0.01)	-0.17 (-0.22, 0.07, -0.02, 0.01)
**Aerosols - indirect and semi-direct**	-	-0.14 ± 0.03	-0.16 ± 0.04
**BC albedo (effective forcing x5)**	-	-0.010 (-0.05)	-0.017 (-0.09)
Net	*-0.32*	*-0.60*	*-0.77*

Additional results

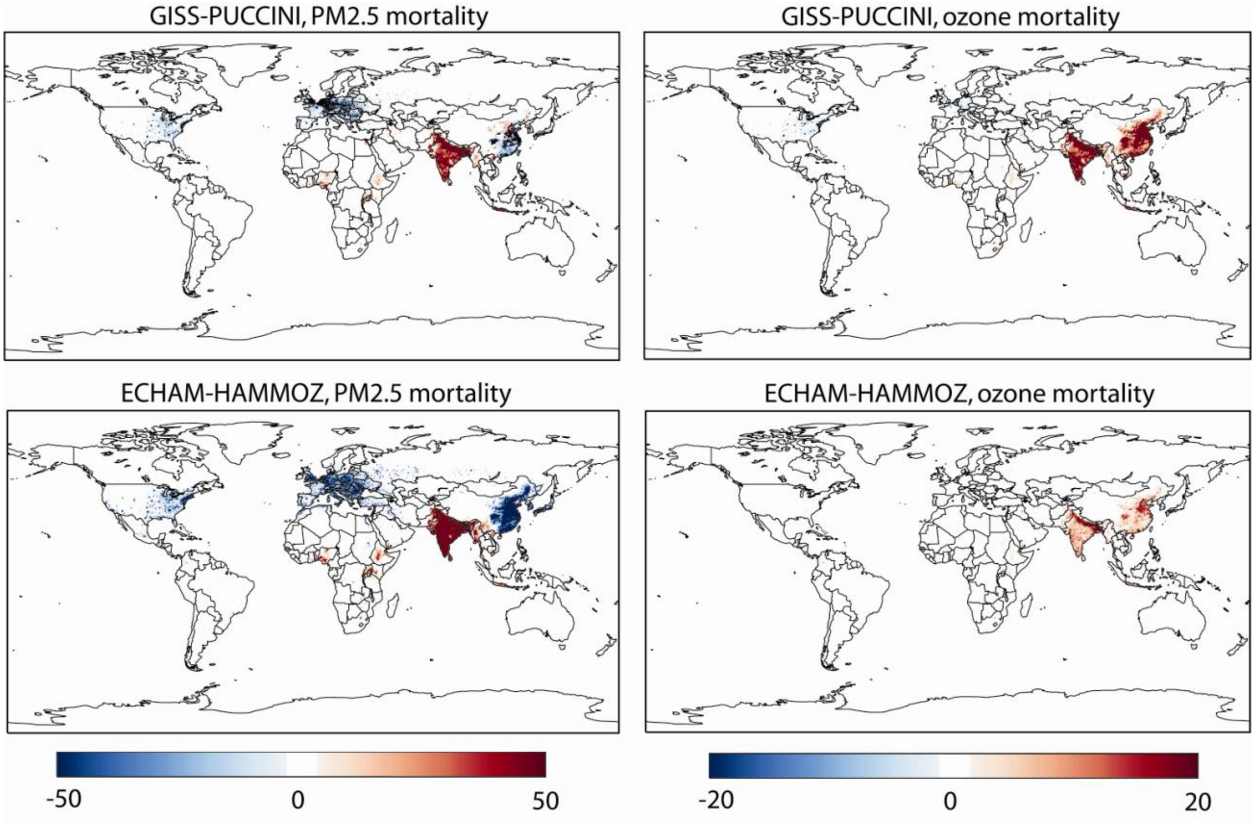

Supplemental Material, Figure 4. Change in estimated annual premature $PM_{2.5}$ cardiopulmonary and lung cancer and ozone respiratory deaths (lives per 1000 km^2) for the 2030 reference scenario relative to 2005, based on 2030 population.

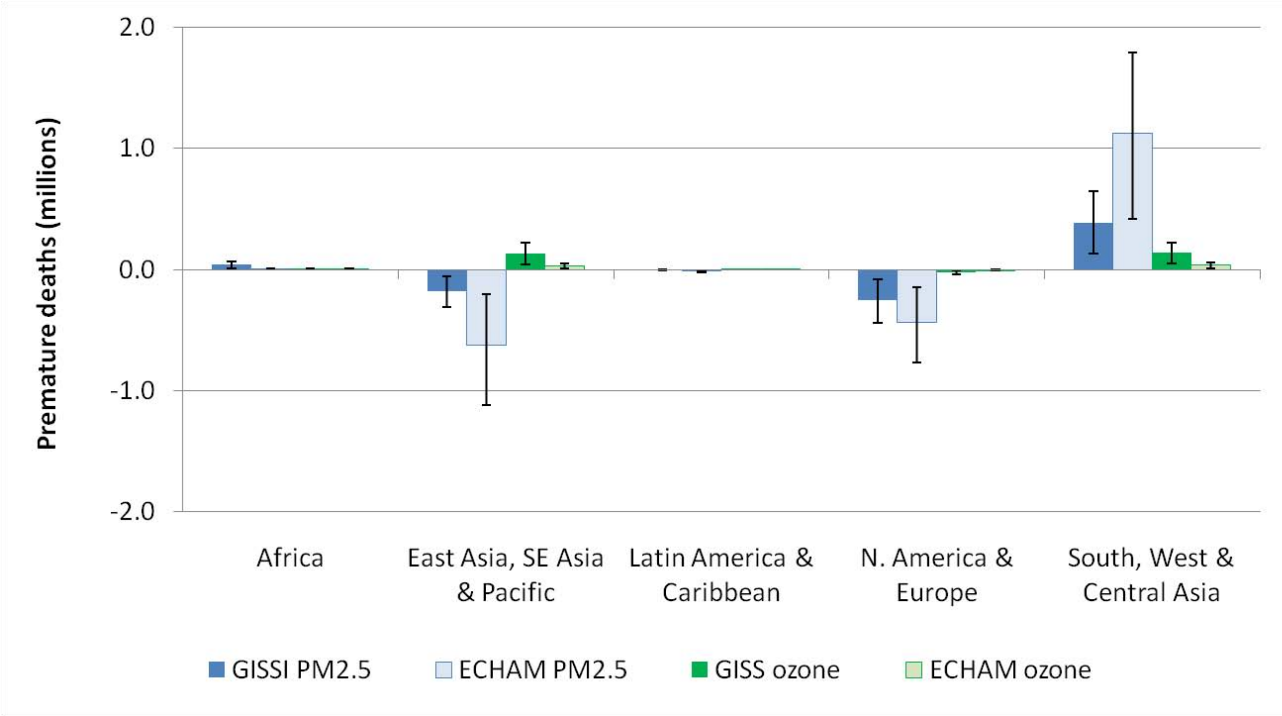

Supplemental Material, Figure 5. Regional change in estimated annual $PM_{2.5}$ cardiopulmonary and lung cancer and ozone respiratory mortality for the 2030 reference scenario relative to 2005, based on 2030 population. Confidence intervals (95%) reflect uncertainty in the CRF only.

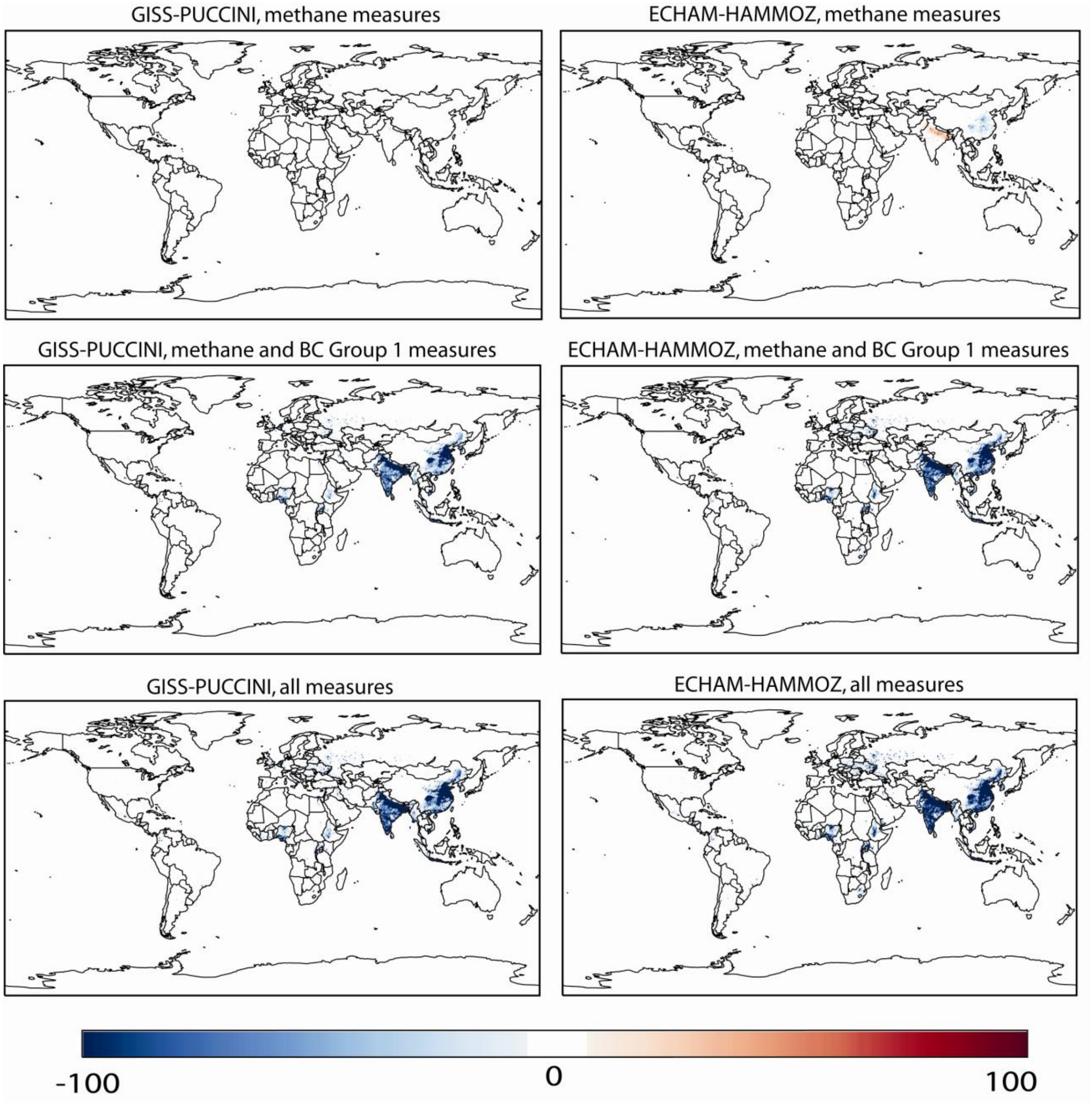

Supplemental Material, Figure 6. Change in estimated annual premature $PM_{2.5}$ cardiopulmonary and lung cancer and ozone respiratory deaths (lives per 1000 km^2) for the successive implementation of methane measures, methane plus BC Group 1 measures, and methane plus BC Group 1 and BC Group 2 measures, relative to the 2030 reference scenario, based on 2030 population.

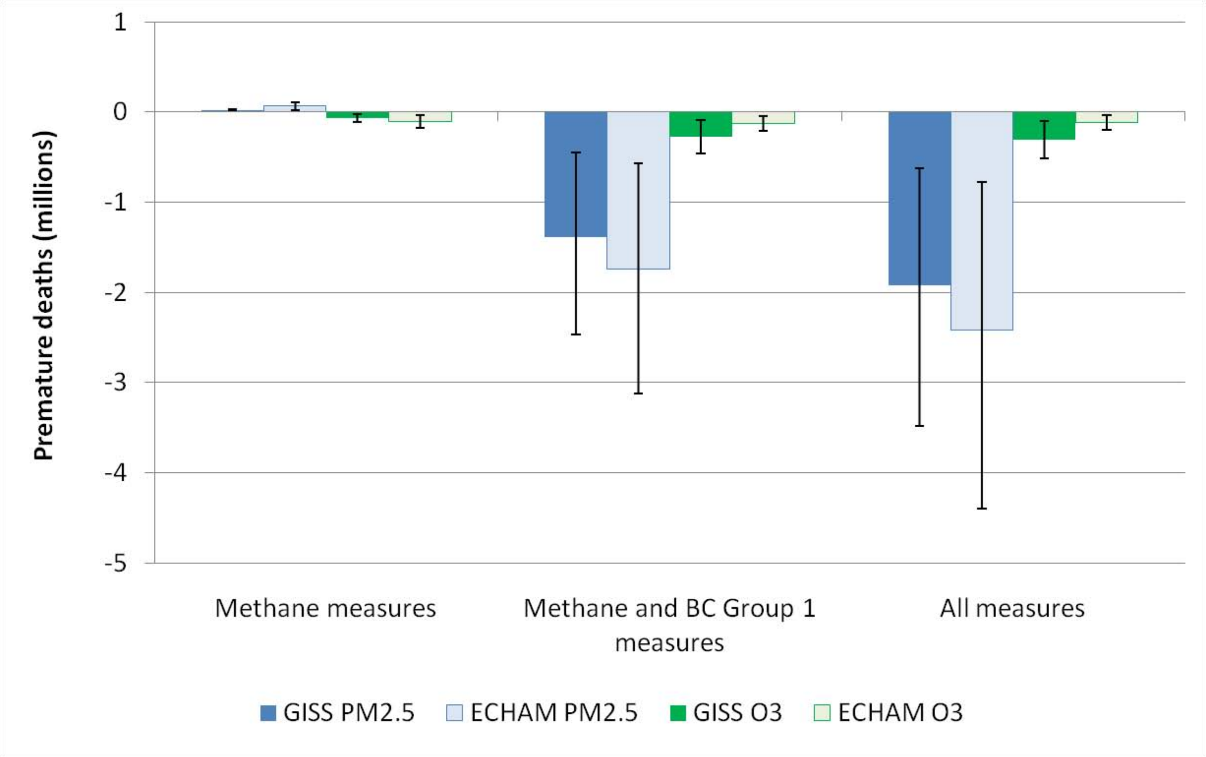

Supplemental Material, Figure 7. Estimated global annual avoided premature $PM_{2.5}$ cardiopulmonary and lung cancer and ozone respiratory deaths for the successive implementation of methane measures, methane plus BC Group 1 measures, and methane plus BC Group 1 and BC Group 2 measures, relative to the 2030 reference scenario, based on 2030 population. Confidence intervals (95%) reflect uncertainty in the CRF only.

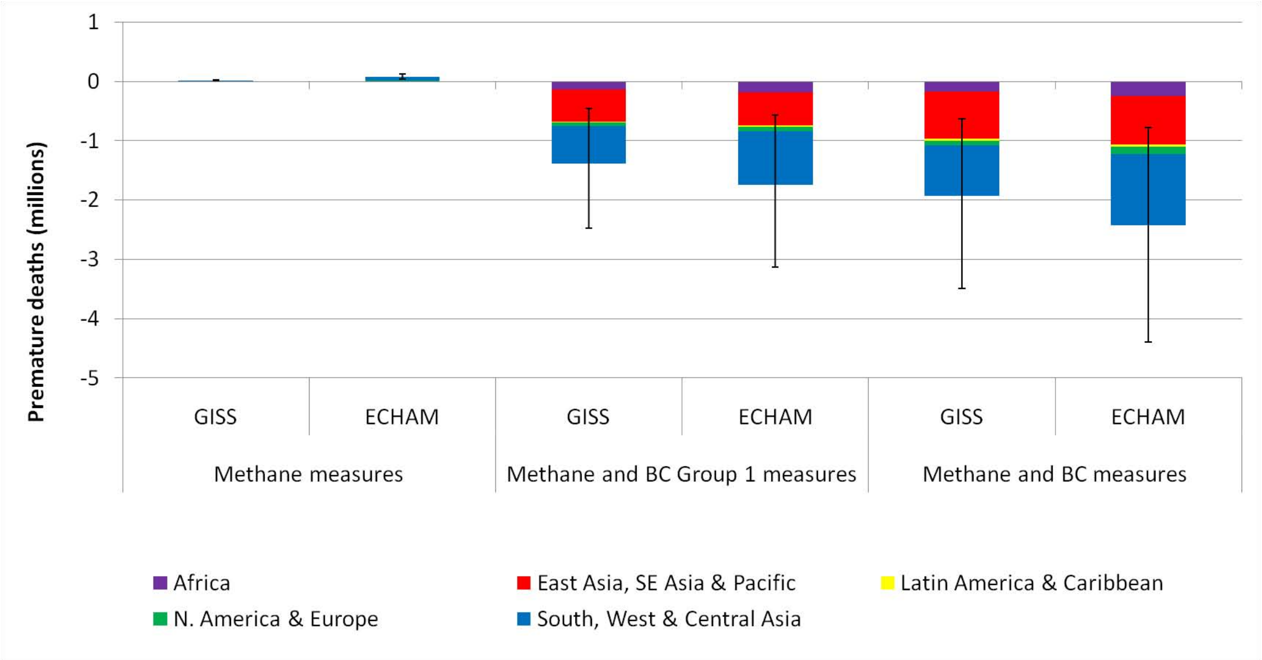

Supplemental Material, Figure 8. Regional change in estimated $PM_{2.5}$ cardiopulmonary and lung cancer mortality for the successive implementation of methane measures, methane plus BC Group 1 measures, and methane plus BC Group 1 and BC Group 2 measures, relative to the 2030 reference scenario, based on 2030 population. Confidence intervals (95%) reflect uncertainty in the CRF only.

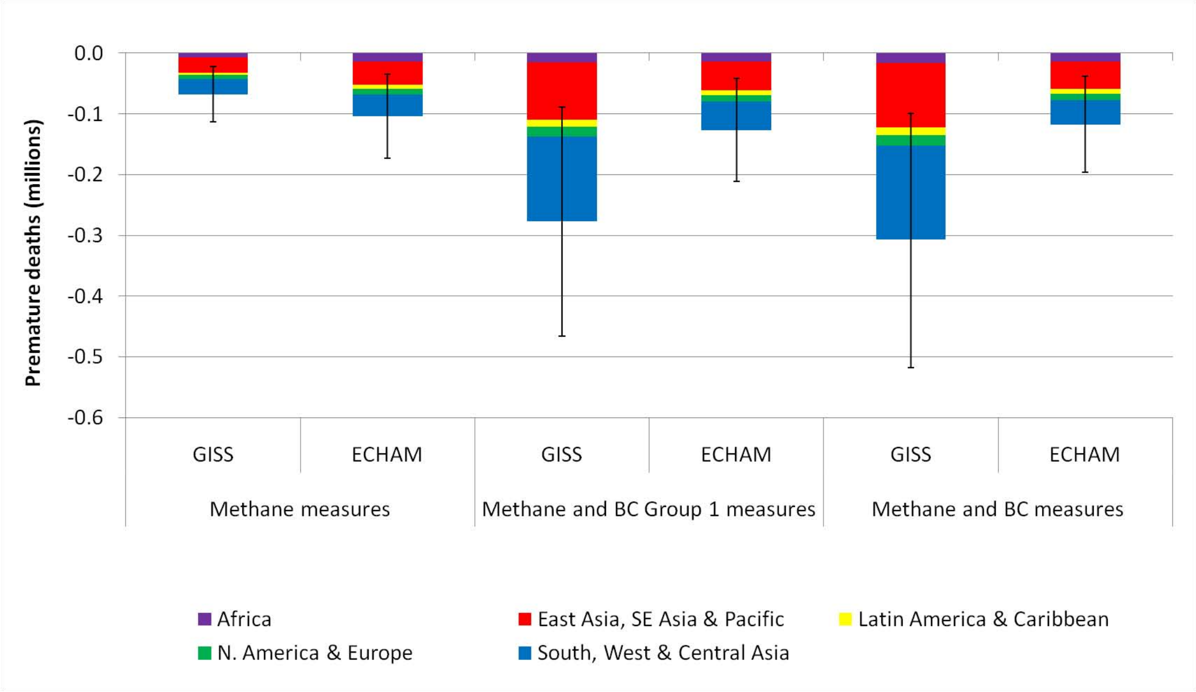

Supplemental Material, Figure 9. Regional change in estimated ozone respiratory mortality for the successive implementation of methane measures, methane plus BC Group 1 measures, and methane plus BC Group 1 and BC Group 2 measures, relative to the 2030 reference scenario, based on 2030 population. Confidence intervals (95%) reflect uncertainty in the CRF only.

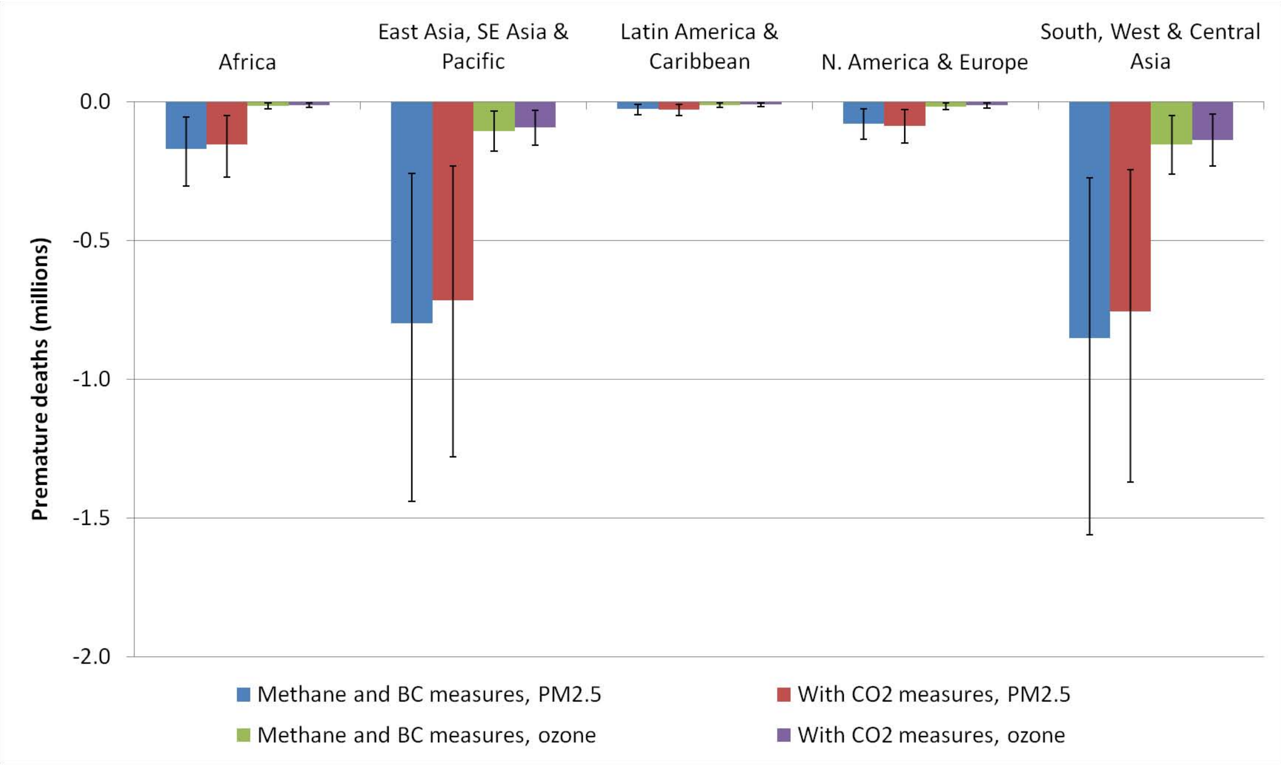

Supplemental Material, Figure 10. Regional change in estimated $PM_{2.5}$ cardiopulmonary and lung cancer and ozone respiratory mortality for the implementation of all methane and BC mitigation measures relative to the 2030 reference scenario and all methane and BC measures with CO_2 mitigation measures included in both the reference and mitigation scenarios, using concentrations simulated by the GISS model and 2030 population. Confidence intervals (95%) reflect uncertainty in the CRF only.

Comparison of results with previous studies

Here we compare results from this study with previous estimates of the health impacts of methane concentration reductions, black carbon emission reductions, and adoption of European vehicle emission standards in developing countries. West et al. (2006) calculated 17,000 avoided cardiopulmonary deaths in 2030 due to a 20% global methane reduction. This estimate is 10% lower than our ozone-mortality response to methane measures after adjusting for the difference in methane reductions (38% vs. 20%, assuming a linear relationship between emissions and concentration), a factor of 2 higher for long-term mortality vs. short-term mortality (Anenberg et al. 2011b), differences in population (9.16 billion vs. 8.4 billion), and a low-concentration threshold of 25 ppb used by West et al. (2006) that gave 2% lower results. Anenberg et al. (2011a) calculated that halving anthropogenic BC and OC globally avoids 157,000 and 1.05 million premature deaths annually in 2002. Scaling these results by the larger emission changes in this study (69% and 79% for BC and OC) and population growth (30% higher in 2030), the adjusted estimate (BC+OC) is 4% higher than our $PM_{2.5}$-mortality response to all measures based on GISS simulations (40% higher using the same RR estimates from Krewski et al. 2009) and 20% lower than the ECHAM-based response. Adopting European vehicle emission standards in developing countries has been estimated to avoid 200,000 premature deaths annually in 2030 (Shindell et al. 2011), ~10% of the health benefits of all measures calculated here using the same GISS model (14% using the same GISS model and CRF from Krewski et al. 2009).

References

Anenberg SC, Talgo K, Arunachalam S, Dolwick P, Jang C, West JJ. 2011a. Impacts of global, regional, and sectoral black carbon emission reductions on surface air quality and human mortality. Atmos Chem Phys 11:7253-7267.

Anenberg SC, West JJ, Horowitz LW, Tong DQ. 2011b. The global burden of air pollution on mortality: Anenberg et al. respond. Environ Health Perspect 119:a158-a159.

Bond TC, and Sun H. 2005. Can reducing black carbon emissions counteract global warming? Environ Sci Technol 39:5921-5926.

Fuglestvedt JS, Shine KP, Bernsten T, Cook J, Lee DS, Stenke A, et al. 2009. Transport impacts on atmosphere and climate: metrics. Atmos Environ 44:4648-4677.

International Energy Agency (IEA). 2009. World Energy Outlook 2009. Paris:International Energy Agency.

Krewski D, Jerrett M, Burnett RT, Ma R, Hughes E, Shi Y, et al. 2009. Extended Follow-up and Spatial Analysis of the American Cancer Society Study Linking Particulate Air Pollution and Mortality. Boston, MA:Health Effects Institute.

Lamarque J-F, Bond TC, Eyring V, Granier C, Heil A, Klimont Z, et al. 2010. Historical (1850-2000) gridded anthropogenic and biomass burning emissions of reactive gases and aerosols: Methodology and application. Atmos Chem Phys 10:7017-7039.

Shindell D, Faluvegi G, Walsh M, Anenberg SC, Van Dingenen R, Muller Z, et al. 2011. Climate, health, agricultural and economic impacts of tighter vehicle-emission standards. Nature Climate Change 1:59-66.

Shindell D, Kuylenstierna JCI, Vignati E, Van Dingenen R, Amann M, Klimont Z, et al. 2012. Simultaneously mitigating near-term climate change and improving human health and food security. Science 335:183-189.

UNEP (United Nations Environment Programme). 2011. Opportunities to limit near-term climate change: An integrated assessment of black carbon and tropospheric ozone and its precursors. Nairobi:United Nations Environment Programme.

West JJ, Fiore AM, Horowitz LW, Mauzerall DL. 2006. Global health benefits of mitigating ozone pollution with methane emission controls. Proceedings of the National Academy of Sciences 103:3988-3993.
